# Interactions between bacterial vaginosis-associated microbiota and *Trichomonas vaginalis* modulate parasite-induced pathogenicity and host immune responses

**DOI:** 10.1186/s13071-025-06996-5

**Published:** 2025-08-14

**Authors:** Shu-Fang Chiu, Ching-Yun Huang, Chien-Yung Chen, Wei-Jane Hsu, Yuan-Ming Yeh, Ya-Wen Shih, Lichieh Julie Chu, Wei-Ning Lin, Kuo-Yang Huang

**Affiliations:** 1 Graduate Institute of Medical Sciences, National Defense Medical Center, Taipei City, 114 Taiwan; 2 Host-Parasite Interactions Laboratory, National Defense Medical Center, Taipei City, 114 Taiwan; 3 Department of Inspection, Taipei City Hospital, Renai Branch, Taipei City, 106 Taiwan; 4 Genomic Medicine Core Laboratory, Chang Gung Memorial Hospital, Linkou, Taoyuan City, 333 Taiwan; 5 School of Nursing, National Taipei University of Nursing and Health Sciences, Taipei City, 112 Taiwan; 6 Graduate Institute of Biomedical Sciences, Chang Gung University, Taoyuan City, 333 Taiwan; 7 Molecular Medicine Research Center, Chang Gung University, Taoyuan City, 333 Taiwan; 8 Department of Surgery, Chang Gung Memorial Hospital, Linkou, Taoyuan City, 333 Taiwan; 9 Graduate Institute of Biomedical and Pharmaceutical Science, Fu Jen Catholic University, New Taipei City, 242 Taiwan; 10 Graduate Institute of Pathology and Parasitology, National Defense Medical Center, No.161, Sec. 6, Minquan E. Rd., Neihu Dist., Taipei City, 114 Taiwan

**Keywords:** *Trichomonas vaginalis*, *Prevotella bivia*, BVB, Adhesion, Host immune response

## Abstract

**Background:**

Trichomoniasis, caused by *Trichomonas vaginalis* (Tv), is the most common nonviral sexually transmitted infection (STI). Bacterial vaginosis (BV) is characterized by a reduction in health-associated *Lactobacillus* and an overgrowth of anaerobes. Both BV-associated bacteria (BVB) and Tv are linked to adverse gynecologic outcomes. Herein, we aimed to investigate whether interactions between vaginal bacterial species and Tv could modulate Tv pathogenicity and Tv-induced host immune responses.

**Methods:**

We established a co-culture system to evaluate the interaction between Tv and various vaginal bacteria, including *Lactobacillus crispatus*, *Escherichia coli*, *Prevotella bivia*, and *Lactobacillus iners*, in the context of polymicrobial infection in ectocervical cells (Ect1). The impact of the interactions between Tv and these bacterial species on Tv adhesion, Tv-induced cytotoxicity in Ect1 cells, and cytokine secretion were assessed. Additionally, the molecular mechanisms governing host inflammation following Tv-bacteria interactions were investigated.

**Results:**

Our in vitro model showed that specific BVB, particularly *P. bivia*, enhanced the expression of Tv *ap65* gene and promoted Tv adhesion to host cells. Additionally, Tv pretreated with *P. bivia* increased cytotoxicity and upregulated IL-6, IL-8, CXCL1, and IP-10 secretion in Ect1 cells. Furthermore, Ect1 cells stimulated with Tv pretreated with *P. bivia* also activated the PI3K, ERK, and p38 MAPK pathways, triggering epithelial-mesenchymal transition (EMT) events. These results demonstrate that this potential pathobiont enhances Tv pathogenicity, highlighting the impact of the vaginal microbiome on host cells during Tv infection*.*

**Conclusions:**

This study significantly advances our understanding of the complex host-bacteria-parasite interactions in the vaginal ecosystem.

**Graphical Abstract:**

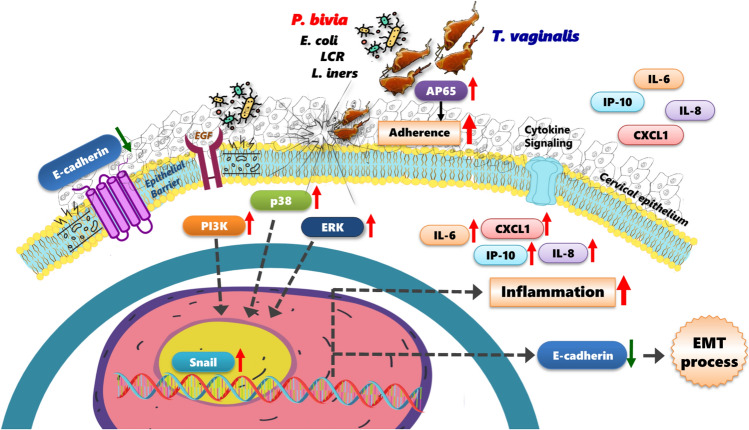

**Supplementary Information:**

The online version contains supplementary material available at 10.1186/s13071-025-06996-5.

## Background

*Trichomonas vaginalis* (Tv), an extracellular protozoan parasite, is recognized as the causative agent of trichomoniasis. According to the World Health Organization, trichomoniasis remains the most prevalent non-viral sexually transmitted infection (STI) globally, with approximately 156 million new cases among adults aged 15–49 in 2020 [1]. Co-infections involving Tv and a variety of other sexually transmitted pathogens are frequently observed in women presenting with dysbiotic vaginal microbiota [2]. Vaginal dysbiosis, characterized by a reduction of lactobacilli and an overgrowth of anaerobic bacteria, such as *Gardnerella vaginalis*, *Atopobium vaginae*, *Prevotella bivia*, is closely linked to bacterial vaginosis (BV) [3, 4]. The interplay between trichomoniasis and BV, along with the associated changes in the cervicovaginal microbial environment, has significant clinical implications, including an increased risk of human immunodeficiency virus (HIV) transmission, various gynecological complications, and even cervical cancer [5]. The underlying mechanism is believed to involve the disruption of the cervicovaginal epithelial barrier, which plays a critical role in protecting against infectious pathogens [6–9].

Adhesion to host cells is a vital step in the establishment of Tv infections [10]. Studies investigating the molecular mechanisms of this adhesion process have identified several key adhesion proteins (APs), including AP65, AP51, AP33, and AP23 [11]. Among these, AP65 is particularly significant for facilitating the binding of Tv to host epithelial cells through ligand-receptor interactions [12]. The interaction between Tv and host cells is not merely superficial, which initiates a contact-dependent mechanism that leads to host epithelial cell death [13]. High-density attachment of Tv to cervicovaginal epithelial cells is associated with increased cytotoxicity, which is crucial for disease pathogenesis. Intriguingly, the presence of lactobacilli, which predominantly inhibit Tv cytoadhesion, provides a protective effect against this cytotoxic process [14]. In contrast to the protective effects exhibited by lactobacilli, recent studies have revealed that Tv, when accompanied by bacteria associated with BV (BVB), elicits synergistic pro-inflammatory responses in human Ect1 cells [15]. Additionally, these investigations have demonstrated that BVB significantly enhances the adherence of Tv to human Ect1 cells. In this study, we used the Ect1/E6E7 cell line to model host–pathogen interactions, highlighting a complex interplay that exacerbates the pathogenesis of these infections [15, 16].

Women with Tv or BV infections within the cervicovaginal mucosa exhibit elevated levels of pro-inflammatory cytokines, such as interleukin-6 (IL-6) and IL-8. These cytokines serve as early indicators of the inflammatory cascade, marking the onset of the host’s inflammatory response to infection [17, 18]. This inflammatory response is orchestrated by various signaling pathways, including mitogen-activated protein kinase (MAPK) and phosphoinositide 3-kinase (PI3K), which are crucial during the early stages of infection in host cells [19, 20]. Numerous studies have demonstrated the pathological effects of IL-6 and IL-8 during Tv infection, particularly in the context of prostate cancer. These cytokines are known to facilitate cell proliferation, migration, and survival, acting through the PI3K and MAPK signaling pathways [21, 22]. Additionally, chemokine (C-X-C motif) ligand 1 (CXCL1) has been implicated in epithelial-mesenchymal transition (EMT), angiogenesis, and the promotion of cancer cell migration and invasiveness, with recent findings highlighting its significant role in both triggering EMT and advancing tumor progression [23, 24]. Interferon-gamma inducible protein 10 kDa (IP-10) has been recognized as a predictive biomarker for BV and bacterial STIs in women infected with HIV, with a remarkable downregulation in individuals diagnosed with BV [25, 26]. Despite robust epidemiological evidence supporting the synergistic relationship between protozoans and bacteria in evading vaginal immunity, the molecular mechanisms underlying the combined effects of Tv and specific bacteria on host immune responses have yet to be elucidated.

Given the frequent coexistence of BVB and Tv in the cervicovaginal environment, this study aimed to investigate the impact of their crosstalk on parasite pathogenesis and host immune responses. A co-culture system was established to explore the interactions between Tv and specific vaginal bacterial species, assessing how these interactions influence Tv adhesion and Tv-induced cytotoxicity in Ect1 cells. Our findings provide the first evidence that the crosstalk between Tv and specific BVB may potentiate Tv-induced host inflammation, initiating a sequence of inflammatory signaling cascades and the EMT process in Ect1 cells. Our model paves the way for further investigation into the complex interactions among Tv, vaginal bacteria, and host within the human vaginal microenvironment, advancing our understanding of how these interactions influence Tv pathogenesis in the host.

## Methods

### Cell culture

A Tv cell line (ATCC 30236) was cultured in YI-S medium at pH 5.8, containing 10% heat-inactivated horse serum and 1% glucose at 37 °C [27]. The growth of parasites was monitored using trypan blue exclusion using a hemocytometer. The human ectocervical cell line Ect1/E6E7 (ATCC CRL-2614^™^) was cultured in Keratinocyte Serum-Free Medium (KSFM)(Gibco, Thermo Fisher Scientific, Inc., Waltham, MA, USA) supplemented with 0.1 ng/mL human recombinant epidermal growth factor, 0.05 mg/mL bovine pituitary extract, and 0.4 mM calcium chloride (44.1 mg/L) [14].

### Bacterial strains and growth condition

Four bacterial species were used in this study: *Lactobacillus crispatus* (LCR), a representative health-associated commensal; *Escherichia coli*, the most common cause of UTIs; *P. bivia*, a common BV-associated species; and *Lactobacillus iners*, a transitional species linked to dysbiosis. *L. crispatus* was cultured on de Man, Rogosa, and Sharpe (MRS) agar, while *L. iners* and *P. bivia* were grown on Anaerobic Blood Agar (CDC). The above bacterial species were cultured at 37 °C under anaerobic conditions using an AnaeroPack^®^ System (Mitsubishi Gas Chemical Co., Tokyo, Japan). *E. coli* was cultured on Columbia agar II, containing 8% whole horse blood in a 5% CO_2_ incubator at 37 °C. For interaction studies with Tv, bacterial colonies were grown for 24–48 h on appropriate agar plates, harvested with a sterile loop, and then resuspended in sterile Dulbecco’s Phosphate-Buffered Saline (DPBS). Bacterial suspensions were diluted to an optical density of 0.5 at 600 nm.

### Tv-bacteria interaction via co-culture assay

The co-incubation of Tv with the aforementioned bacterial species was performed as previously described [28]. Briefly, Tv (5 × 10^5^ cells/mL) and vaginal bacteria were co-cultured in 1 mL of KSFM (Gibco, Thermo Fisher Scientific, Inc., Waltham, MA, USA) at a ratio of 1:10 for 6 h at 37 °C. Following incubation, these organisms were recovered by centrifugation at 200 × *g* for 5 min at 4 °C, then washed three times with PBS containing 200 μg/mL gentamicin to eliminate bacteria. To verify the effectiveness of bacterial removal, post-wash samples were plated on MRS, anaerobic blood agar, or Columbia blood agar and incubated for 48 h. The absence of visible colony formation confirmed that gentamicin treatment combined with repeated washing effectively eliminated viable bacteria prior to downstream assays Subsequently, Tv was washed twice with PBS to remove residual antibiotics [29]. Parallel incubations of Tv alone were performed as controls. After co-incubation, the growth of Tv was evaluated using the trypan blue exclusion assay.

### RNA extraction and cDNA synthesis

After the co-incubation of Tv with various bacterial species, total RNA was extracted from Tv using the TRIzol Plus RNA Purification System (Invitrogen, USA). The RNA concentration and purity (A260/A280 ratio) were measured using a NanoDrop ND-1000 spectrophotometer (Thermo Fisher Scientific, Waltham, MA, USA). To synthesize cDNA, 1 μg of RNA was reverse-transcribed using the First-Strand Synthesis System (Invitrogen, USA). Briefly, RNA was mixed with dNTP, Oligo (dT), and DEPC-treated water, then incubated at 65 °C for 5 min. The reaction mix was supplemented with 10X RT Buffer, 0.1 M DTT, RNaseOUT, and SuperScript^™^ II reverse transcriptase, followed by incubation at 50 °C for 50 min. The reaction was terminated at 85 °C for 5 min, after which RNase H was added, and the mixture was incubated at 37 °C for 20 min to degrade residual RNA.

### Reverse transcription-PCR

Reverse transcription PCR (RT-PCR) was conducted using the GoTaq^®^ Colorless Master Mix (Promega, USA) according to the manufacturer’s instructions [30, 31]. cDNA was synthesized from 1 μg of total RNA extracted from Tv. Primers are listed in Table S1. The RT-PCR products were mixed with a DNA staining reagent (Novel Juice, GeneDireX^™^, USA), separated on a 1.7% agarose gel, and visualized under UV light. Band intensities were quantified using ImageJ software (NIH, USA). To normalize gene expression levels, *β*-tubulin was used as an internal control. The relative expression of each target gene was calculated by determining the ratio of the band intensity of the target gene to that of *β*-tubulin (target/*β*-tubulin).

### Tv adherence assay

To evaluate the impact of Tv-bacteria interactions on parasite adherence to Ect1 cells, Tv pretreated with four bacterial species was stained using a CellTracker^™^ Blue CMAC fluorescent probe (Invitrogen, Carlsbad, CA, USA) according to the manufacturer’s instructions. Briefly, Ect1 cells were seeded on 12-mm coverslips in 24-well plates and cultured to confluence in appropriate medium at 37 °C with 5% CO₂. Labeled Tv (1 × 10^5^ cells in 0.5 mL of complete KSFM, stained with 10 μM CellTracker^™^ Blue CMAC) was added to confluent Ect1 monolayers. After co-incubation for 30, 60, and 120 min, coverslips were gently washed with PBS, fixed with 4% formaldehyde, and mounted on slides using Mowiol polyvinyl alcohol mounting medium to prevent photobleaching. Fluorescently labeled parasites adherent to Ect1 cells were counted using ImageJ software. For quantification, 15 randomly selected fields per coverslip were recorded at 10 × magnification using fluorescence microscopy. Each experimental condition was performed in triplicate, representing three independent biological replicates. Results are expressed as the percentage of adherent parasites relative to the total number of parasites added per coverslip [32].

### Cell viability assay

To determine the impact of Tv-bacteria interactions on Ect1 cell viability, Ect1 cells were seeded on 24-well plates and cultured in complete KSFM medium supplemented with 5% fetal bovine serum (FBS). After 24 h of incubation, the medium was replaced with serum-free KSFM. Tv (1 × 10^5^ cells/mL) pretreated with four bacterial species was added to the Ect1 cells (1 × 10^5^ cells/mL) and co-incubated for 30, 60, and 120 min. Cell viability was assessed using the Cell Counting Kit-8 (CCK-8) (Sigma-Aldrich, USA). The CCK-8 reagent was added to each well, followed by incubation for 30 min. Absorbance was measured at 450 nm using an ELISA Reader to determine cell viability.

### Enzyme-linked immunosorbent assay (ELISA)

To detect the production of inflammatory cytokines from Ect1 cells stimulated with Tv pretreated with various bacterial species, Tv (1 × 10^5^ cells/mL) was added to the Ect1 cells (1 × 10^5^ cells/mL) and co-incubated for 30, 60, and 120 min. Following incubation, culture supernatants were collected and stored at −80 °C. The levels of IL-6, IL-8, IP-10, and CXCL1 were measured using ELISA kits (R&D System, USA) and quantified by detecting absorbance at 450 nm with an ELISA reader.

### Immunoblotting

To determine whether Ect1 cells stimulated with Tv pretreated with various bacterial species activates the PI3K and MAPK signaling pathways, as well as the EMT process, Ect1 cells (1 × 10^5^cells per well) were seeded in 6-well plates and co-incubated with Tv for 30, 60, and 120 min. After incubation, cells were lysed using RIPA buffer (Bio-Future, Taiwan) with a protease inhibitor cocktail (Bio-Future, Taiwan) on ice for 30 min. Whole cell lysates (10 µg per sample) were separated by 12% sodium dodecyl sulfate polyacrylamide gel electrophoresis (SDS-PAGE) and transferred to polyvinylidene fluoride (PVDF) membranes. After blocking with 5% milk, the membranes were incubated with primary antibodies overnight at 4 °C. The following primary antibodies were used for immunoblotting: antibodies against phosphor-PI3K, phosphor-ERK, phosphor-p38 MAPK, *E*-cadherin, Snail (1:1000; Cell Signaling Technology Inc., Danvers, MA, USA), and *β*-Actin (1:2000; Cell Signaling Technology Inc., Danvers, MA, USA). Following three washes with tris-buffered saline with 0.1% tween (TBST, Bio-Future, Taiwan), the membranes were incubated with horseradish peroxidase-conjugated goat anti-rabbit IgG (1:5000; Cell Signaling Technology, Beverly, MA, USA) for 90 min. Bound secondary antibodies were detected using an enhanced chemiluminescence (ECL) substrate (Merck Millipore, USA). Band intensities of each target protein were quantified using ImageJ software (NIH, USA) and normalized to the corresponding *β*-actin band from the same lane. Normalized values from three independent experiments were used for statistical analysis.

### Statistical analysis

All statistical analyses were conducted using GraphPad Prism version 5.01 (GraphPad Software, San Diego, CA). Quantitative data are expressed as mean ± SD from three independent experiments. Significant differences between groups were determined using unpaired two-tailed Student’s *t*-tests. A *p*-value of < 0.05 was considered statistically significant.

## Results

### Tv interacted with specific BVB affects the growth and virulence of parasites

**Fig. 1:**
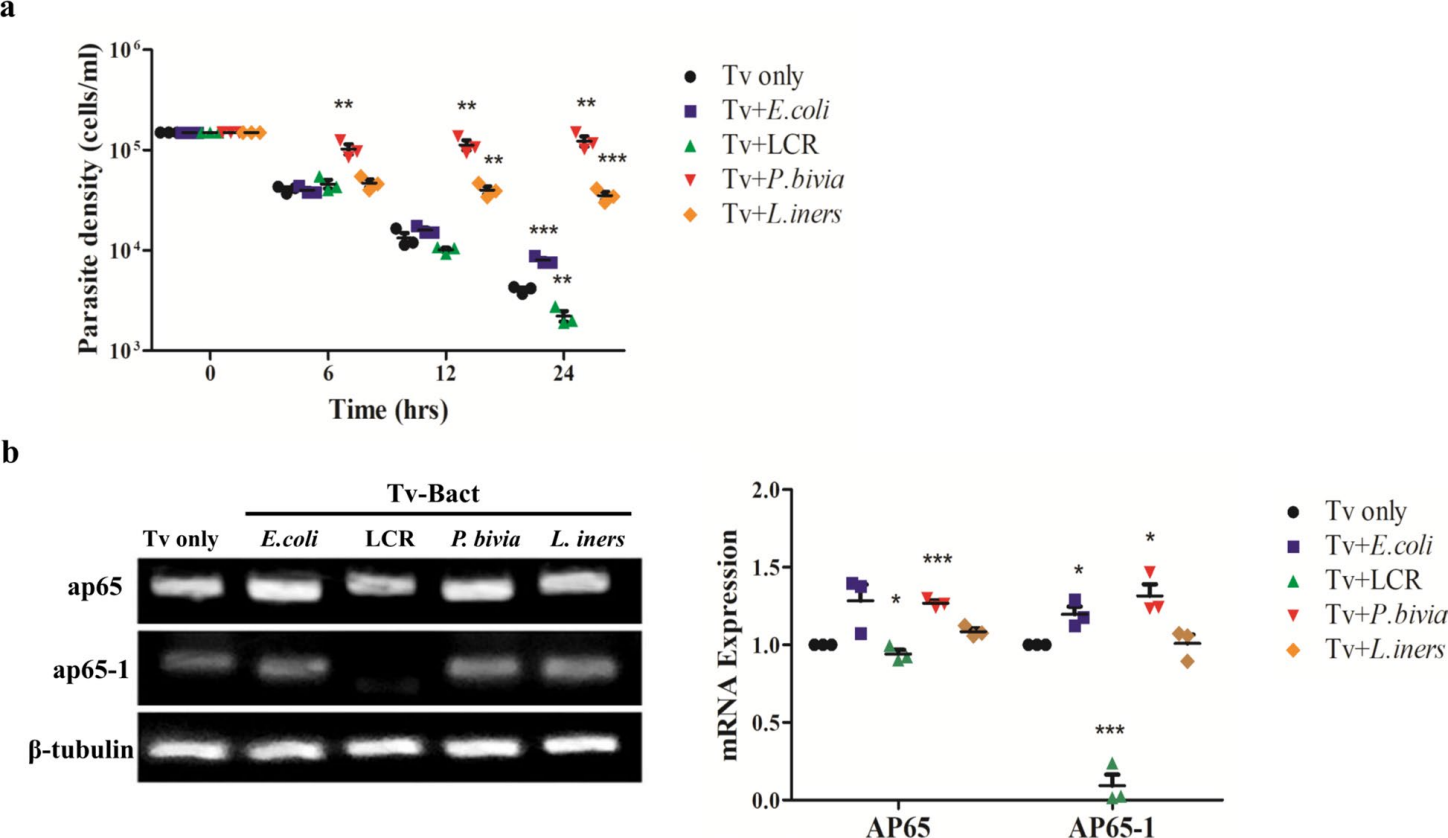
The impact of interactions between Tv and vaginal bacterial species on Tv growth and *ap65*-related gene expression. **a** Tv was co-incubated with *E. coli*, *L. crispatus* (LCR), *P. bivia*, and *L. iners* at a ratio of 1:10 for 0, 6, 12, and 24 h. After co-incubation, the organisms were centrifugated and washed with PBS containing gentamicin. Tv cultivation alone (Tv only) served as the control. The growth of Tv pretreated with each bacterial species was monitored by trypan blue exclusion using a hemocytometer. **b** The mRNA expression levels of *ap65*-related genes in Tv upon interaction with these bacterial species for 6 h were examined by RT-PCR. RT-PCR products were separated by electrophoresis on a 1.7% agarose gel and band intensities were quantified using ImageJ software. The mRNA expression levels of *ap65*-related genes were calculated and normalized to *β*-tubulin. The Tv only group served as the baseline reference for comparative analysis. Quantitative data are presented as mean ± SD from three independent experiments. *P < 0.05; **P < 0.01; ***P < 0.001

The vaginal microbiome analysis has been recently investigated in Tv-infected patients, indicating that the relative abundance of *P. bivia* was significantly enriched [3]. The influence of the interactions between vaginal bacterial species and Tv on parasite growth was also verified. Following a similar approach, we established a co-culture system to assess the effect of various bacteria on Tv growth [28]. Four distinct bacterial species were used in this study: LCR, a representative health-associated commensal; *E. coli*, the most common cause of urinary tract infections (UTIs); *P. bivia*, a common BV-associated species; and *L. iners*, a transitional species linked to a dysbiotic state. Tv was co-incubated with these bacterial species at a ratio of 1:10, and the growth of Tv was subsequently monitored at 0, 6, 12, and 24 h. The Tv-only control group displayed limited proliferation after 6 h (Fig. 1a), likely owing to nutrient constraints in the serum-free co-culture system. This suggests that 6-h co-incubation period is optimal to capture early microbial interactions, minimizing confounding effects from prolonged co-culture. Notably, co-incubation with *P. bivia* consistently enhanced Tv proliferation across all time points compared with Tv cultivation alone. These findings suggest that the interaction between Tv and specific BVB promotes Tv growth within the vaginal microenvironment*.*

The expression of Tv adhesion protein AP65 serves as a biomarker for evaluating the pathogenicity of Tv. AP65 is a multigene family, with *ap65-1* as one of its members, sharing similar structure and function with other family proteins [33]. Iron has been shown to upregulate the transcription initiation and expression level of *ap65-1*, thereby regulating the expression of Tv adhesion proteins [12, 34]. To examine the impact of the interactions between vaginal bacteria and Tv on parasite pathogenicity, we analyzed the expression levels of *ap65*-related genes in Tv following co-incubation with these bacterial species. Notably, the expression of *ap65* gene was upregulated in Tv pretreated with *P. bivia* for 6 h, while both *ap65* and *ap65-1* genes were upregulated in Tv pretreated with *E. coli* and *P. bivia*, compared with Tv cultivation alone (Tv only) (Fig. 1b). In contrast, co-incubation with LCR markedly inhibited the expression of these genes, and no significant difference was observed in Tv after interaction with *L.iners*. These results demonstrate that the *ap65*-related genes are significantly upregulated in Tv upon interaction with *P. bivia*, suggesting that specific BVB can enhance the virulence of the parasite.

### Tv pretreated with specific vaginal bacteria reduces Ect1 viability and enhances the adherence of Tv to Ect1 cells

**Fig. 2:**
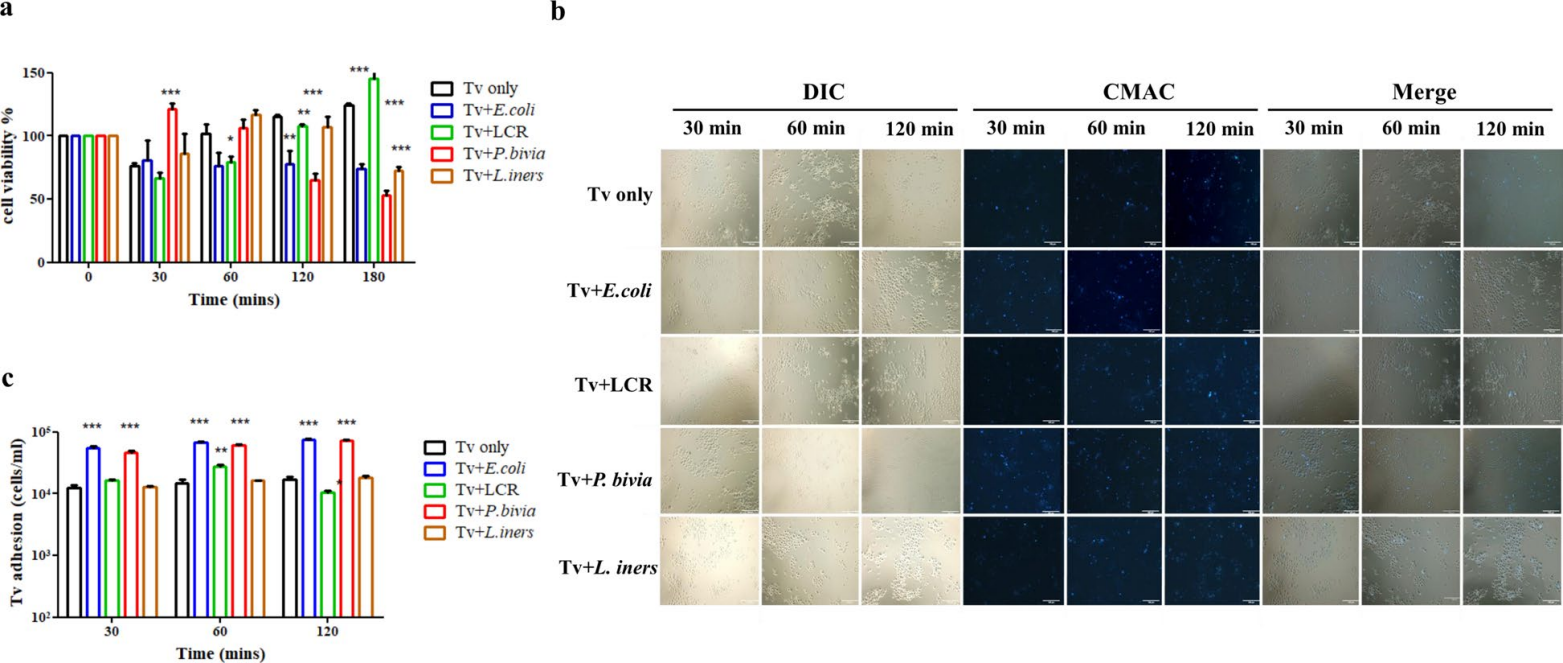
The impact of interactions between Tv and vaginal bacterial species on Ect1 cell viability and parasite adhesion. Tv was co-incubated with *E. coli*, *L. crispatus* (LCR), *P. bivia*, and *L. iners* at a ratio of 1:10 for 6 h. **a** After co-incubation of Tv with various bacteria, Ects were exposed to Tv for 0, 30, 60, 120, and 180 min. The viability of Ects was assessed using the CCK-8 assay. **b** Representative brightfield, fluorescence (CMAC-labeled), and merged images showing the adhesion of Tv to Ect1 cells at 30, 60, and 120 min. **c** Quantification of Tv adhesion to Ect1 cells at 30, 60, and 120 min using ImageJ software. Tv cultured alone (Tv only) served as the control. Scale bar = 100 μm. Quantitative data are presented as mean ± SD from three independent biological experiments. *P < 0.05; **P < 0.01; ***P < 0.001

The mucosal epithelium acts as a natural physical barrier against microbial invasion. The crosstalk between Tv and BVB might affect the integrity of the human cervicovaginal mucosal barrier induced by Tv infection [15]. We next investigated whether the pre-incubation of Tv with different vaginal bacterial species, as previously described, affects the viability of Ect1 cells. When Ect1 cells were exposed to Tv pre-incubated with *P. bivia*, an initial increase in Ect1 viability was observed at 30 min, followed by a decrease after 120 min of interaction (Fig. 2a). After prolonged co-incubation, the viability of Ect1 cells progressively decreased, from 66.6% at 120 min to 52.7% at 180 min after interaction with Tv pretreated with *P. bivia*. Similarly, both *E. coli*- and LCR-pretreated Tv caused a statistically significant reduction in Ect1 cell viability after 120 min of interaction compared with the Tv only group. These findings suggest that Ect1 cells exposed to Tv pretreated with specific vaginal bacteria undergo substantial cellular damage after 120 min of interaction. Hence, a co-incubation period of up to 120 min was selected for subsequent experiments.

Next, we investigated whether the interaction between these bacteria and Tv affects the adhesion of Tv to Ect1 cells. Tv pretreated with each bacterial species was labeled with Cell Tracker Blue CMAC and subsequently co-incubated with Ect1 cells for different time intervals. The adherence of CMAC-labeled Tv to Ect1 cells was quantified, revealing that Tv pretreated with *E. coli* and *P. bivia* significantly increased the adherence to Ect1 cells after 30 min of interaction, and this effect persisted for at least 120 min (Fig. 2b, c). Additionally, the adhesion of Tv pretreated with LCR enhanced after 60 min of interaction with Ect1, but significantly decreased after 120 min. In contrast, no significant change in adhesion was observed for Tv pretreated with *L. iners*. Together, Tv pretreated with *P. bivia* and *E. coli* exhibited enhanced adhesion to Ect1 cells over a 2-h period compared with the control group (Tv only). This result correlates with the upregulation of *ap65*-related genes and the reduced viability of Ect1 cells, suggesting that Tv-induced contact-dependent damage to Ect1 cells is exacerbated by specific vaginal bacteria.

### Tv pretreated with specific BVB enhances the secretion of pro-inflammatory cytokines

**Fig. 3:**
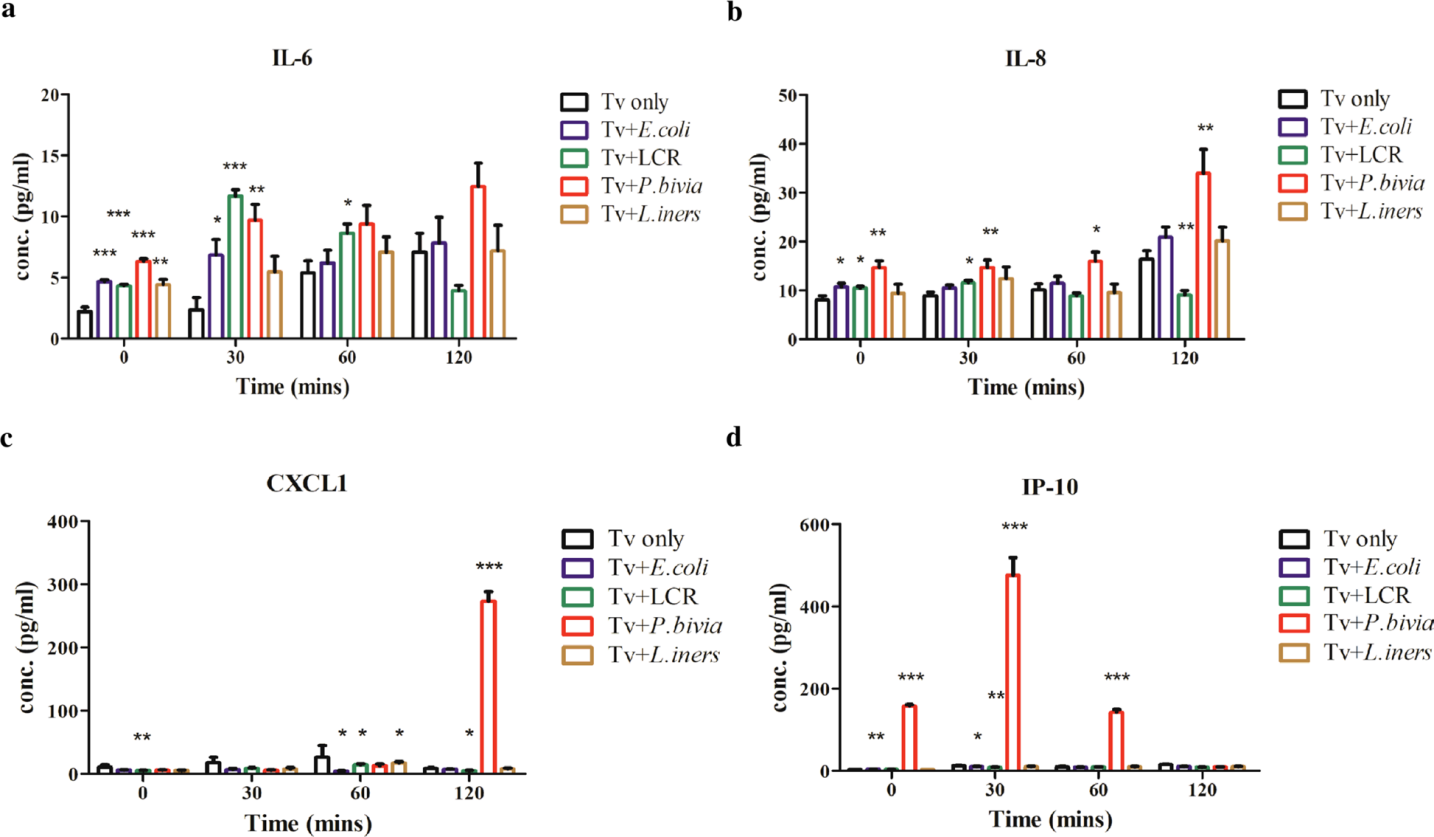
Tv pretreated with specific BVB increases the secretion of the inflammatory cytokines in Ect1 cells. Tv was co-incubated with *E. coli*, *L. crispatus* (LCR), *P. bivia*, and *L. iners* at a ratio of 1:10 for 6 h. After co-incubation, Ect1 cells were exposed to Tv for 0, 30, 60, and 120 min, and the levels of IL-6 **a**, IL-8 **b**, CXCL1 **c**, and IP-10 **d** were measured using ELISA. Tv cultivation alone (Tv only) served as the control. Quantitative data are presented as mean ± SD from three independent experiments. *P < 0.05; **P < 0.01; ***P < 0.001

Human Ect1 cells are known to produce pro-inflammatory cytokines in response to Tv and BVB [15]. Additionally, bacteria associated with Tv infection have been shown to enhance the pro-inflammatory response to Tv-specific antigens [16]. The interaction between BVB and Tv has been shown to induce the secretion of the pro-inflammatory cytokine IL-6 from Ect1 cells [15]. Moreover, there is evidence linking BV to the presence of monocytes in mucosal secretions in women infected with Tv, which is associated with elevated levels of IL-8 and IP-10 [35]. To evaluate the effects of the interaction between Tv and vaginal bacterial species on cytokine production from Ect1 cells, Ect1 cells were co-incubated with Tv pretreated with the aforementioned bacterial species for 0, 30, 60, and 120 min. Baseline cytokine levels in Ect1 cells at the 0-min time point remained low across most groups, except for *P. bivia*-pretreated Tv, which induced slightly elevated cytokine levels. After 30 min of interaction, IL-6 secretion from Ect1 cells stimulated with Tv pretreated with *E. coli*, LCR, and *P. bivia* was significantly increased compared with the Tv only group (Fig. 3a). Additionally, IL-8 secretion from Ect1 cells stimulated with *P. bivia*-pretreated Tv was markedly elevated at all incubation time points compared with the Tv only group (Fig. 3b). In contrast, Ect1 cells stimulated with LCR-pretreated Tv reduced the production of IL-8 after prolonged stimulation.

CXCL1 has been shown to play a key role in inflammation [24]. The secretion of CXCL1 was significantly upregulated when Ect1 cells were stimulated with Tv pretreated with *P. bivia* after 120 min of interaction (Fig. 3c). Additionally, the production of IP-10 was markedly increased in Ect1 cells stimulated with *P. bivia*-treated Tv after 30–60 min of interactions, followed by a subsequent decrease with prolonged stimulation (Fig. 3d). Collectively, these findings suggest that the interactions between Tv and specific vaginal bacterial species, particularly *P. bivia,* may enhance the parasite’s immunomodulatory activity, leading to increased secretion of pro-inflammatory cytokines by host cells.

### Tv pretreated with specific bacteria augments the activation of PI3K, ERK, and p38 MAPK signaling pathways in Ect1 cells

**Fig. 4:**
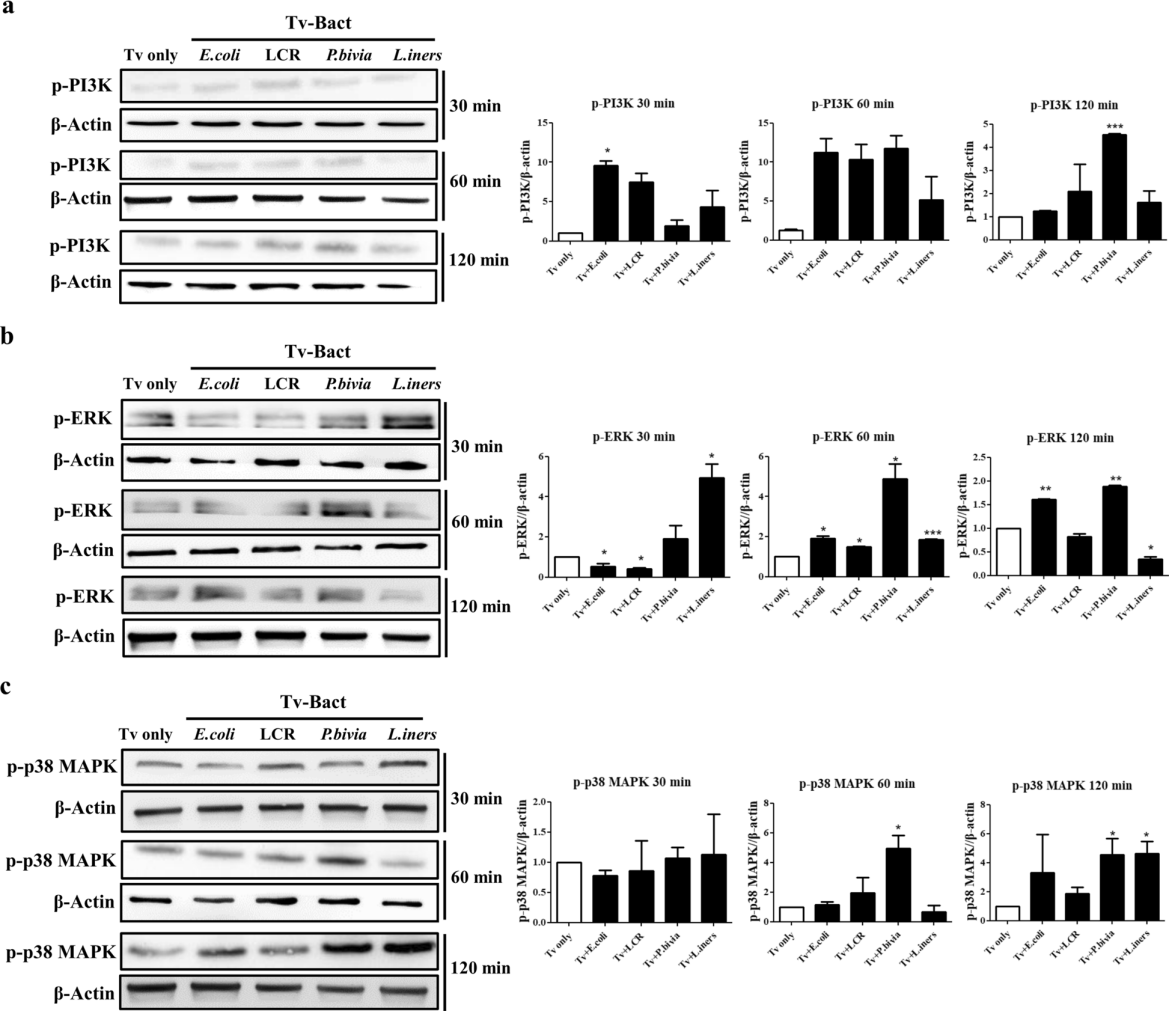
Tv pretreated with specific bacterial species enhances the activation of PI3K and MAPK signaling pathways in Ect1 cells. Tv was co-incubated with *E. coli*, *L. crispatus* (LCR), *P. bivia*, and *L. iners* at a ratio of 1:10 for 6 h. After co-incubation, Ect1 cells were exposed to Tv for 30, 60, and 120 min, and Ect1 cell lysates were subsequently analyzed by western blotting using antibodies specific for phosphorylated PI3K (*p*-PI3K) **a**, ERK (*p*-ERK) **b**, and p38 MAPK (*p*-p38 MAPK) **c**. Tv cultivation alone (Tv only) served as the control. *β*-actin was used as a loading control for western blotting. Band intensities of each target protein were quantified using ImageJ software and normalized to the corresponding *β*-actin band from the same lane. Quantitative data are presented as mean ± SD from three independent experiments. *P < 0.05; **P < 0.01; ***P < 0.001

Several chemokines and pro-inflammatory cytokines are markedly upregulated following Tv infection, potentially triggering an array of signaling cascades that evoke inflammation [36, 37]. The PI3K and MAPK pathways play crucial roles in regulating the production of pro-inflammatory cytokines in Tv-infected cervical mucosal epithelial cells [38]. We then investigated whether Tv pretreated with various vaginal bacteria could modulate the activation of the PI3K and MAPK pathways in Ect1 cells. Notably, phosphorylation of PI3K was significantly enhanced in Ect1 cells stimulated with *E. coli-*pretreated Tv for 30 min and with *P. bivia-*pretreated Tv for 120 min compared with the Tv only group (Fig. 4a). Additionally, phosphorylation of extracellular signal-regulated kinase (ERK) in Ect1 cells stimulated with Tv pretreated with *E. coli* and *P. bivia* significantly increased after 60 and 120 min of interaction, while Ect1 cells stimulated with *L. iners*-pretreated Tv exhibited increased ERK phosphorylation after 30 and 60 min of interaction, followed by a decrease at 120 min (Fig. 4b). Moreover, phosphorylation of p38 MAPK was markedly activated in Ect1 cells stimulated with *P.bivia-*pretreated Tv for 60 min and 120 min and with *L. iners-*pretreated Tv for 120 min (Fig. 4c). Conversely, no significant activation of the PI3K and p38 MAPK pathways was observed when Ect1 cells were stimulated with LCR-pretreated Tv. Collectively, these results suggest that the PI3K and MAPK signaling pathways are involved in mediating the inflammatory cascades in Ect1 cells stimulated with Tv pretreated with various vaginal bacterial species. Although the activation kinetics of these pathways differs, they may contribute to the alteration of pro-inflammatory cytokine secretion in Ect1 cells, influencing the host immune response during Tv infection.

### P. bivia enhances the ability of Tv to induce EMT in Ect1 cells

**Fig. 5:**
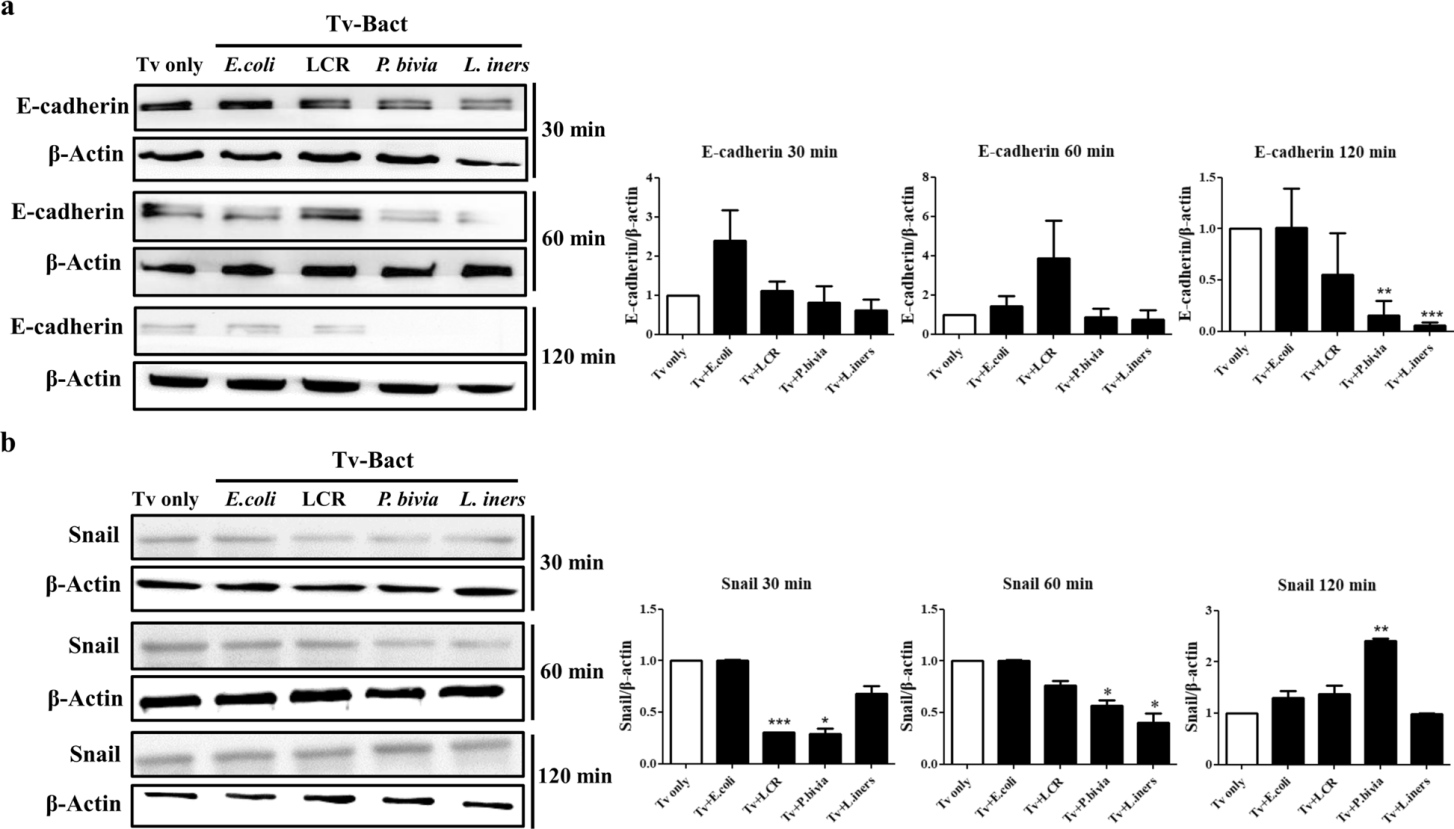
*P. bivia* enhances the ability of Tv to induce EMT of Ect1 cells. Following the co-culture of Tv and various bacterial species at a ratio of 1:10 for 6 h, Ect1 cells were incubated with Tv for 30, 60, and 120 min. Ect1 cell lysates were analyzed by western blotting using antibodies specific for *E*-cadherin **a** and Snail **b**. Tv cultivation alone (Tv only) served as the control. *β*-actin was used as a loading control for western blotting. Band intensities of each target protein were quantified using ImageJ software and normalized to the corresponding *β*-actin band from the same lane. Quantitative data are presented as mean ± SD from three independent experiments. *P < 0.05; **P < 0.01; ***P < 0.001

EMT is characterized by the breakdown of cell-to-cell or cell-to-extracellular matrix (ECM) adhesion in the polarized epithelial tissues. Under normal conditions, *E*-cadherin expression is typically high in cervical tissue; however, its downregulation is a hallmark of EMT during pathological states [39]. Conversely, Snail, a transcription factor that promotes EMT, is upregulated in pathological conditions [40]. To investigate the impact of interactions between Tv and vaginal bacterial species on EMT in Ect1 cells, the expression of EMT markers was determined by western blot analysis. Notably, *E*-cadherin expression was significantly reduced in Ect1 cells stimulated with *P. bivia*-pretreated Tv for 120 min, while Snail expression was markedly upregulated under the same conditions (Fig. 5a, b). In contrast, no significant changes in both *E*-cadherin downregulation and Snail upregulation were observed in Ect1 stimulated with Tv pretreated with other bacterial species. These findings indicate that EMT is specifically induced in Ect1 cells stimulated with *P. bivia*-pretreated Tv, suggesting that interaction between Tv and this specific BVB enhances the parasite’s ability to promote EMT in host cells.

## Discussion

Microbial synergism involves the cooperative activities between two or more microorganisms, leading to an enhanced disease phenotype. The microbiota may harbor indigenous pathobionts that contribute to disease progression by manipulating host immune responses [41–43]. Previous studies have reported that cooperative interactions between Tv and BVB can significantly influence the course of infection [15, 16]. Additionally, endosymbionts of Tv, such as *Mycoplasma hominis* and Trichomonasvirus (TVV), have been shown to directly impact the parasite’s virulence and immunopathogenesis [44]. For instance, Tv and *M. hominis* engage in co-metabolism of arginine, which promotes microbial growth and reduces the availability of free nitric oxide, potentially impairing the ability of macrophages to efficiently eliminate parasites [45]. If specific vaginal bacteria function as pathobionts for Tv, they may enhance the parasite’s infectivity by facilitating its nutrition, growth, immune evasion, and virulence. The adherence of Tv to host cells is a critical step in infection and a major determinant of its virulence, influencing its cytotoxic effects on host cells [10, 13, 46]. In our experimental model, Tv was pre-incubated with vaginal bacteria prior to exposure to Ect1 cells. This approach was designed to mimic early-stage interactions between Tv and vaginal microbiota, reflecting the scenario in which Tv encounters bacteria before contacting the epithelial surface. Therefore, we focused on elucidating the impact of the interactions between Tv and associated vaginal bacteria, highlighting the potential synergetic roles of specific bacteria in modulating Tv pathogenesis and host immune responses. This in vitro model based on the Ect1 cell line allows for consistent and reproducible evaluation of host-parasite interactions. Further validation using primary ectocervical epithelial cells will help to better reflect in vivo conditions.

The significance of an intact mucosal layer is underscored by its role in preventing pathological disorders associated with inflammatory diseases, where disruption of the epithelial barrier can lead to severe mucosal inflammation. Our findings provide robust evidence that damage to host cells by Tv infection is modulated through the crosstalk between Tv and specific vaginal bacterial species. *P. bivia* has been shown to contribute to the production of polyamines, which are involved in cell growth regulation [47]. Additionally, polyamine metabolism can supply up to 10% of the energy required for Tv growth in complex media [48]. Hence, the enhancement of Tv growth upon interaction with *P. bivia* (Fig. 1a) may be attributed to increased polyamine metabolism provided by the specific BVB. Previous reports have demonstrated that Tv infection results in cervical epithelial cell death via a strictly contact-dependent mechanism [13, 49, 50]. We herein showed that the interactions between Ect1 and Tv pretreated with *E. coli* and *P. bivia* promotes the adhesion of parasite to Ect1 and heightened host cell cytotoxicity (Fig. 2). This effect is partially linked to the upregulation of Tv *ap65*-related gene expression induced by *E. coli* and *P. bivia* (Fig. 1b). However, an initial increase in Ect1 cell viability was observed in response to *P. bivia*-pretreated Tv (Fig. 2a). Tv AP65 shares homology with hydrogenosomal malic enzyme, which possesses decarboxylating activity and is also involved in polyamine metabolism [34, 51–53]. Hence, the role of *P. bivia*-driven polyamine metabolism in modulating Tv growth and Ect1 viability within the vaginal ecosystem warrants further investigation.

Recent studies have highlighted the critical role of Tv-derived peptidases, particularly cysteine proteases (CPs), in mediating host cell cytotoxicity and compromising epithelial barrier integrity. TvCP39 and TvMP50 have been implicated in the degradation of junctional proteins, such as *E*-cadherin and occludin, as well as in the induction of host cell death via proteolytic mechanisms [54–59]. In our study, the downregulation of *E*-cadherin (Fig. 5a) and the modulation of MAPK signaling (Fig. 4), particularly in the *P. bivia*-pretreated group, may reflect the activation of such protease-dependent pathogenic mechanisms. Although we did not directly assess peptidase activity, these findings support the hypothesis that Tv-bacteria interactions possibly enhance parasite virulence by influencing proteolytic pathways. Future studies could investigate the expression and enzymatic activity of TvCP39, TvMP50, and other virulence-associated peptidases upon interaction with various bacterial species.

The adhesion of Tv to vaginal epithelial cells is pivotal in the pathogenesis of trichomoniasis, facilitating the production of pro-inflammatory cytokines such as IL-8, CCL2, and IL-6 [49, 60, 61]. Previous studies have reported elevated levels of IL-1β and IL-8 in women co-infected with BV and Tv infections [62]. In this study, Ect1 cells stimulated with Tv pretreated with specific vaginal bacteria, especially *P. bivia*, showed significantly enhanced production of pro-inflammatory cytokines, including IL-6, IL-8, CXCL1, and IP-10, which may contribute to the pathogenesis of cervical diseases in the female reproductive system. IL-6 and IL-8 play critical roles in the early inflammatory response to infections of genital tract epithelial cells [63, 64], as evidenced by the marked secretion of these cytokines in Ect1 cells exposed to Tv pretreated with *P. bivia* in our study. Intriguingly, we report for the first time that CXCL1 and IP-10 are highly secreted in Ect1 upon interaction with Tv pretreated with the BVB *P. bivia*, emphasizing the need to further investigate individual bacterial species as drivers of immune imbalances within the disturbed vaginal microbiome. CXCL1 attracts several immune cells, especially neutrophils, to the site of infection and thus regulates the immune responses [20, 65]. Additionally, CXCL1 has been shown to be upregulated in cervical cancer [23], triggering EMT [66]. These findings suggest that *P. bivia* is likely to be involved in the development of cervical cancer in the context of Tv infection. Additionally, we observed a significant increase in IP-10 secretion in Ect1 cells exposed *P. bivia*-pretreated Tv, which may facilitate the recruitment of endocervical CD4 + T cells to the mucosa, potentially increasing susceptibility to HIV infection [25, 67–69]. Therefore, we purpose that the interaction between Tv and *P. bivia* may enhance the risk of HIV acquisition. Collectively, our findings highlight the need for further investigation into the role of *P. bivia* in the development of cervical cancer and in promoting HIV susceptibility after Tv infection.

We found that Tv pretreated with *P. bivia* enhances the activation of PI3K, ERK, and p38 MAPK in Ect1 cells within 2 h of interaction. Notably, the activation kinetics of PI3K, ERK, and p38 MAPK in Ect1 cells exposed to Tv pretreated with various vaginal bacteria exhibited distinct patterns. These pathways have been implicated in the inflammatory response of the Tv-infected cervicovaginal mucosal epithelium [38]. Additionally, PI3K and MAPK signaling pathways are known to contribute to EMT under inflammatory conditions [17, 70–73]. However, we did not elucidate the exact mechanisms by which Tv, in conjunction with specific vaginal bacteria, disrupt the Ect1 cell barrier. The molecular pathways driving the increased production of pro-inflammatory cytokines and the EMT events in Ect1 cells following Tv-bacteria interactions remain unclear. Further investigation is required to clarify whether the activation of signaling pathways, such as PI3K and MAPK, plays a role in these processes. Additionally, a limitation of our study is the omission of endosymbionts of Tv, such as *M. hominis* and TVVs, which may alter the host immune response associated with Tv infection [44, 45].

Previous investigations into the pathogenesis of trichomoniasis have mainly focused on host-parasite interactions without considering the associated vaginal microbiota. In earlier work, we demonstrated the impact of specific vaginal bacteria on Tv growth [28]. In this study, we provide insights into the roles of vaginal bacterial species in promoting Tv pathogenesis and modulating host immune responses, highlighting the importance of microbial interactions in disease progression. Vaginal metagenome analysis of South African women revealed that a high abundance of *P. bivia* is associated with a 19-fold increase in the likelihood of a pro-inflammatory vaginal cytokine profile and approximately a 13-fold higher risk of acquiring HIV [74]. We herein confirmed the causative role of this Gram-negative anaerobe as a pathobiont of Tv and demonstrated the impact of their interactions on altering host immune response via activating the PI3K and MAPK pathways. Future studies could further investigate the mechanisms of the synergetic effects of *P. bivia* on Tv pathogenesis by conducting large-scale transcriptomic and proteomic analyses, potentially identifying novel genes or proteins involved in parasite virulence and host immune modulation.

## Conclusions

**Fig. 6:**
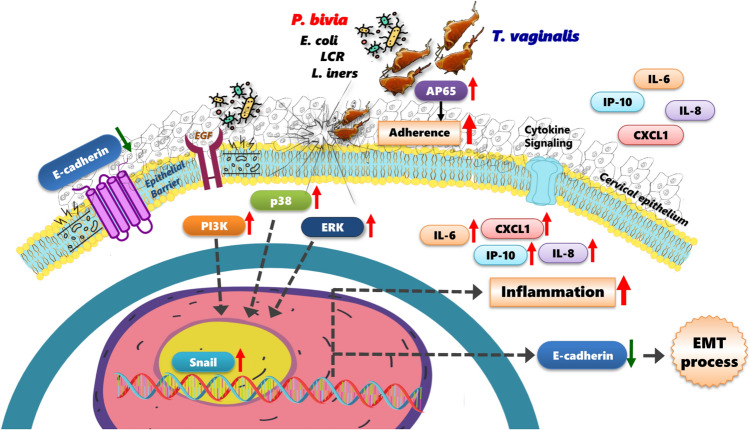
Schematic model illustrating the crosstalk between Tv and various vaginal bacterial species in modulating parasite pathogenesis and host immune responses. Specific BVB, such as *P. bivia*, acts as pathobionts for Tv, enhancing Tv adherence to Ect1 cells and exacerbating Ect1 cytotoxicity. Additionally, Tv pretread with *P. bivia* activates the PI3K, ERK, and p38 MAPK signaling pathways, as well as EMT in Ect1 cells, leading to increased secretion of the pro-inflammatory cytokines, which may contribute to disease progression in the female reproductive system

Collectively, our findings demonstrate that vaginal bacteria from a dysbiotic microbiome enhance the pathogenic potential of Tv. Specifically, certain BVB, such as *P. bivia*, modify Tv’s capacity to adhere to host cells, exacerbating host cytotoxicity, promoting EMT, and driving pro-inflammatory cytokine production. The interaction between Tv and specific vaginal bacteria further modulates the host immune response via activating inflammatory signaling cascades, potentially resulting in the secretion of multiple cytokines that may contribute to disease progression in the female reproductive system (Fig. 6). Further research is needed to comprehensively elucidate the mechanisms by which specific vaginal microbes synergistically influence the pathogenesis of trichomoniasis and other important Tv-associated diseases, such as HIV and cervical cancer. Our findings establish a clear link between specific vaginal bacterial species and Tv, demonstrating their potential impact on host cells. This work significantly advances our understanding of the complex crosstalk between vaginal bacteria, Tv, and host cells, providing new insights into the dynamic interactions occurring within the vaginal ecosystem during Tv infection.

## Supplementary Information

Additional file 1.

## Data Availability

Data supporting the main conclusions of this study are included in the manuscript.

## Abbreviations

Tv: *Trichomonas vaginalis*
STI: Sexually transmitted infection
BV: *bacterial vaginosis*
BVB: BV-associated bacteria
Ect1: Ectocervial cells
EMT: Epithelial-mesenchymal transition
HIV: Human immunodeficiency virus
APs: Adhesion proteins
IL-6: Interleukin-6
MAPK: Mitogen-activated protein kinase
PI3K: Phosphoinositide 3-kinases
CXCL1: Chemokine (C-X-C motif) ligand 1
IP-10: Interferon-gamma inducible protein 10 kDa
LCR: *Lactobacillus crispatus*
UTIs: Urinary tract infections
ERK: Extracellular signal-regulated kinase
ECM: Extracellular matrix
TVV: Trichomonasvirus
KSFM: Keratinocyte Serum-Free Medium
MRS: De Man, Rogosa, and Sharpe
DPBS: Dulbecco’s Phosphate-Buffered Saline
FBS: Fetal bovine serum
CCK-8: Cell Counting Kit-8
ELISA: Enzyme-linked immunosorbent assay
SDS-PAGE: Sodium dodecyl sulfate polyacrylamide gel electrophoresis
TBST: Tris-buffered saline with 0.1% tween
CP: Cysteine protease
